# Association between arsenic exposure and soluble thrombomodulin: A cross sectional study in Bangladesh

**DOI:** 10.1371/journal.pone.0175154

**Published:** 2017-04-11

**Authors:** M. M. Hasibuzzaman, Shakhawoat Hossain, Md. Shofikul Islam, Atiqur Rahman, Adiba Anjum, Faruk Hossain, Nayan Chandra Mohanto, Md. Rezaul Karim, Md. Mominul Hoque, Zahangir Alam Saud, Hideki Miyataka, Seiichiro Himeno, Khaled Hossain

**Affiliations:** 1 Department of Biochemistry and Molecular Biology, University of Rajshahi, Rajshahi, Bangladesh; 2 Department of Applied Nutrition and Food Technology, Islamic University, Kushtia, Bangladesh; 3 Laboratory of Molecular Nutrition and Toxicology, Faculty of Pharmaceutical Sciences, Tokushima Bunri University, Tokushima, Japan; University of Kentucky, UNITED STATES

## Abstract

Chronic exposure to arsenic is associated with increased morbidity and mortality from cardiovascular disease (CVD); however, plausible biomarker for early prediction and the underlying mechanism of arsenic-related CVD have not yet been clearly understood. Endothelial dysfunction plays a central role in the development of CVD. We hypothesized that endothelial damage or dysfunction is an important aspect and may be an early event of arsenic-related CVD. Soluble thrombomodulin (sTM) in serum is thought to be a specific and stable marker for endothelial damage or dysfunction. This study was designed to evaluate the association between chronic exposure to arsenic and sTM among human subjects in arsenic-endemic and non-endemic rural areas in Bangladesh. A total of 321 study subjects (217 from arsenic-endemic areas and 104 from a non-endemic area) were recruited. Subjects’ arsenic exposure levels (i.e., drinking water, hair and nail arsenic concentrations) were measured by Inductively Coupled Plasma Mass Spectroscopy. The subjects’ serum sTM levels were quantified by immunoassay kit. The average sTM levels of the subjects in arsenic-endemic and non-endemic areas were 4.58 ± 2.20 and 2.84 ± 1.29 (ng mL^-1^) respectively, and the difference was significant (*p*<0.001). Arsenic exposure levels showed a significant (water arsenic: *r*_*s*_ = 0.339, *p*<0.001, hair arsenic: *r*_*s*_ = 0.352, *p*<0.001 and nail arsenic: *r*_*s*_ = 0.308, *p*<0.001) positive associations with sTM levels. Soluble TM levels were higher in the higher exposure gradients if we stratified the subjects into tertile groups (low, medium and high) based on the arsenic concentrations of the subjects’ drinking water, hair and nails. Finally, increased levels of sTM were negatively correlated with high density lipoprotein cholesterol (HDL-C), and positively correlated with intercellular adhesion molecule-1 (ICAM-1) and vascular cell adhesion molecule-1 (VCAM-1). Results of this study show that chronic exposure to arsenic has mild to moderate association with sTM levels.

## Introduction

Arsenic is a major threat to the public health in many countries including Bangladesh and West Bengal of India where arsenic poisoning has been termed as the largest mass poisoning in the history of human civilization [1]. Tens of thousands of people suffer from arsenic toxicity and additional 80–100 million of people are at risk of toxicity because they consume arsenic through drinking water at greater than the permissive limit set by the World Health Organization (WHO) [2]. Several epidemiological studies have found the link between chronic arsenic exposure and almost all forms of cardiovascular disease (CVD) [3–19].

Endothelial function plays a central role in vascular homeostasis. However, endothelial dysfunction is a major physiopathological mechanism that leads to the development of coronary artery disease, and other atherosclerotic diseases [20]. The presence of endothelial dysfunction can be considered as a clinical syndrome that is associated with and predicts an increased rate of cardiovascular diseases [21,22]. Endothelial dysfunction is present at all stages of atherosclerosis-related diseases [23]. Endothelial damage or dysfunction causes an imbalance between vasoconstriction and vasodilation and initiates a number of events related to atherosclerosis including increased endothelial permeability, platelet aggregation, leukocyte adhesion and generation of cytokines [24].

Thrombomodulin (TM) is an integral membrane glycoprotein and high-affinity receptor for thrombin on the endothelial cell surface, and it has been implicated in the endothelial regulation of fibrinolysis and coagulation [25]. Cell bound form of TM binds with thrombin resulting in the neutralization of pro-coagulant actions of thrombin. This receptor engagement enhances anti-coagulant and anti-inflammatory properties of clotting inhibitory protein C through its activation [25,26]. TM is widely distributed on the endothelial cells in arteries, veins, capillaries, and lymphatics in all organs and tissues except the brain [27,28]. After proteolytic cleavage of TM from the injured or damaged endothelial cell surface, soluble TM (sTM) is found in blood and its concentration is thought to reflect the degree of endothelial damage [29,30]. Soluble TM is a promising biomarker for CVD and in the treatment of risk factors associated with CVD [23]. CVD is a major cause of chronic arsenic exposure-related morbidity and mortality [31,32], however, underlying mechanism and potential biomarker for early prediction of the arsenic-related CVD has not yet been clearly established. We, therefore, designed the present study to evaluate the association of chronic exposure to arsenic with sTM in relation to circulating molecules of CVD risk among human subjects in arsenic-endemic and non-endemic rural areas in Bangladesh.

## Materials and methods

### Ethics statement

This study was approved by the Institutional Animal, Medical Ethics, Biosafety and Biosecurity Committee (IAMEBBC) of the Institute of Biological Sciences, University of Rajshahi, Bangladesh (#21/320-IAMEBBC/IBSc). All human subjects who participated in this study gave their written consent. All sorts of confidentialities and the rights of study subjects were strictly maintained. Written consent was also taken from the study subjects. Water specimens were collected from the tube wells that were identified by the study subjects used for their drinking purposes. The study individuals themselves identified their drinking water sources (tube wells), and gave their written consent to participate in this study. Therefore, it was not required to take additional specific permissions for collecting water samples. This research has been conducted in the rural areas of Bangladesh which were not restricted or protected areas declared by any agencies of the country. No specific permissions were required for conducting this research in the study areas and on the study subjects selected for this study.

### Study areas and subjects

The arsenic-endemic and non-endemic areas and study subjects were selected as described [18,19,33–37]. Arsenic-endemic study areas were selected from the northwest region of Bangladesh that included Marua in Jessore, Dutpatila, Jajri, Vultie and Kestopur in Chuadanga and Bheramara in Kushtia districts. Chowkoli, a village in Naogaon district with no history of arsenic contamination was selected as the non-endemic area. Adults (18–60 years old) who had lived for at least the last 5 years in the arsenic-endemic and non-endemic areas were recruited for this study.

We attempted to match, as much as possible the age, sex and socioeconomic parameters of the subjects between the arsenic-endemic and non-endemic areas. The ratios of endemic and non-endemic subjects were approx. 2:1, and the male to female ratios in the endemic and non-endemic areas were also approx. 1:1. The subjects in both the arsenic-endemic and non-endemic areas were villagers, and the socioeconomic parameters such as occupation, monthly income and education levels were very closely matched among the areas.

Pregnant and lactating women, individuals who were hepatitis B-positive, and individuals with a history of drug addiction, chronic alcoholism, prescription for hepatotoxic or anti-hypertensive medications, malaria, kala azar (leishmaniasis) or hepatic, renal or cardiac diseases were excluded from the study. An interview of each subject was carried out by the trained members of our research team who visited each household and used a standard questionnaire. Information obtained from the interview included the sources of water for drinking and daily house hold uses, water consumption history, socioeconomic status, occupation, food habit, cigarette smoking habits, alcohol intake, personal and family medical history, histories of diseases, physiological complications, previous physician’s reports, and body mass index (BMI).

### BP measurement

The WHO standard protocol for measuring BP was used. After the subject had rested for ≥20 min, both systolic and diastolic blood pressures (SBP and DBP) were measured three times with a mercury sphygmomanometer with the subject sitting. SBP and DBP were defined at the first-and fifth-phase Korotkoff sounds, respectively. The average of three measurements was used for the analysis. Hypertension was defined as an SBP value of ≥140 mm Hg and a DBP value of ≥90 mm Hg on three repeated measurements.

### Collection of blood and serum

All study subjects were requested to fast overnight (10–12 h), and fasting blood samples (5–7 ml) were collected from each individual by venipuncture into blood collection tubes. The blood samples were left at room temperature for 30 min for clotting, and were subsequently centrifuged at 1200 × g for 20 min. The serum supernatant was then taken and stored at –80°C.

### Water collection and arsenic analysis

Water samples were collected from the tube wells which the subjects used as a primary source of drinking water, as described [33]. The water samples from the tube wells were collected in acid washed container after the well was pumped for 5 min. The total arsenic concentration in water samples was determined by inductively coupled plasma mass spectroscopy (ICP-MS, HP-4500, Agilent Technologies, Kanagawa, Japan) after the addition of a solution of yttrium (10 ppb in 1.0% nitric acid) as an internal standard for the ICP-MS analysis. The accuracy of the ICP-MS determination of the water arsenic concentrations was confirmed by using 'River water' (NMIJ CRM 7202-a No.347; National Institute of Advanced Industrial Science and Technology, Japan) as a certified reference material. The average value (mean ± SD) of arsenic in the 'River water' determined in triplicate by an ICP-MS analysis was 1.06 ± 0.04 μg/L (reference value, 1.18 μg/L).

### Hair and nail collections and arsenic analysis

Hair and toe nails of the subjects were collected and washed as described [33]. The washed samples were allowed to dry at 60°C overnight and then digested with concentrated nitric acid using a hot plate at 70°C for 15 min and 115°C for 15 min. After cooling, the samples were diluted with 1.0% nitric acid containing yttrium (10 ppb). The concentrations of arsenic and yttrium in these samples were determined by ICP-MS. All samples were determined in triplicate and the average values were used. The accuracy of the arsenic measurement was verified by using the CRM "human hair" (GBW09101, Shanghai Institute of Nuclear Research Academia Sinica, China). The average value of arsenic in "human hair" determined in triplicate followed by an ICP-MS analysis was 0.61 ± 0.12 μg/g (reference value, 0.59 μg/g).

### Measurement of sTM

Serum sTM were measured using commercially available thrombomodulin human enzyme-linked immunoassay kits (Abcam, Cambridge, UK) according to the manufacture’s protocols. A micro-plate reader (Mikura Ltd. UK) was used for the measurement of color development. All standards and samples were analyzed in duplicate. The intra and inter assay coefficients of variations (CVs) were maximum 10%.

### Statistical analyses

The statistical analyses were conducted with the Statistical Package for the Social Sciences (SPSS ver. 21.0, SPSS, Chicago, IL). A *p*-value <0.05 was considered significant. The normality of the distribution of variables was verified by a Q–Q plot. Because of the skewed distributions of the arsenic exposure metrics, we used log-transformed values for the statistical analysis. The differences in descriptive characteristics, arsenic exposure levels and other characteristics between the residents of the arsenic-endemic and non-endemic areas were analyzed by an independent sample *t*-test for continuous variables and the Chi-square test for categorical variables. A nonparametric Kruskal-Wallis test was used to analyze the differences in inter-quartile range (IQR) between the subjects in the arsenic-endemic and non-endemic areas.

Spearman correlation coefficient test was used to evaluate the correlations of arsenic exposure metrics (water, hair and nail arsenic concentrations) with sTM levels. For the analysis of dose-dependent associations, the subjects were stratified through frequency test into low, medium and high exposure groups based on the concentrations of arsenic in the drinking water, hair and nails. Serum sTM levels in the low, medium and high exposure groups were analyzed by one-way ANOVA followed by Bonferroni multiple comparison tests. We performed multiple linear regression analyses to examine the effects of age, sex, BMI, smoking and hypertension on the association between arsenic exposure metrics and sTM levels. To determine the interaction effect between arsenic exposure and hypertension on sTM levels we performed univariate regression analyses. Finally Associations of sTM levels with circulating levels of high density lipoprotein cholesterol (HDL-C), intercellular adhesion molecule-1 (ICAM-1) and vascular cell adhesion molecule-1 (VCAM-1) were evaluated by Spearman correlation coefficient test.

## Results

### Descriptive characteristics of the study subjects

Table 1 summarizes the descriptive characteristics of the study subjects in the arsenic-endemic (n = 217) and non-endemic areas (n = 104). Of the 321 subjects, 170 were males and 151 were females. The ratio of arsenic-endemic and non-endemic subjects was approx. 2:1, and the male-to-female ratios in both the endemic and non-endemic areas were approx. 1:1. The average ages of the study subjects in arsenic-endemic and non-endemic areas were 37.54 ± 11.73 and 35.02 ± 10.38 years, respectively. The mean BMI of the study subjects in the arsenic-endemic and non-endemic areas were 20.50 **±** 3.11 and 21.27 **±** 2.74 kg/m^2^, respectively. Since we attempted to match the subjects’ age, sex and socioeconomic parameters (occupation, monthly income and education) between the two study groups in arsenic-endemic and non-endemic areas, no significant differences were observed in those parameters between the two groups. Most of the male subjects were farmers, and most of the female subjects were housewives. We did not identify any female smokers, as generally Bangladeshi women do not smoke. None of the subjects drank alcohol because of the social and religious restriction on alcohols. The levels of DBP and SBP of the subjects in the arsenic-endemic areas were significantly (p<0.001) higher than those of the subjects in the non-endemic area, as we reported [18]. Accordingly, the percentage of hypertensive subjects was also higher in the arsenic-endemic areas than those of the subjects in non-endemic area. Average concentrations of arsenic in the drinking water, hair and nails of the subjects in the arsenic-endemic areas were approx. 74, 17 and 7 times higher, respectively, than those of the subjects in the non-endemic area. The ranges of arsenic concentrations in the drinking water, hair and nails of the study subjects were 0.03–546.00 μg L^-1^, 0.02–37.24 μg g^-1^ and 0.15–37.42 μg g^-1^, respectively. Average (mean ± SD) sTM levels of the subjects in the arsenic-endemic and non-endemic areas were 4.58 ± 2.20 and 2.84 ± 1.29 ng mL^-1^, respectively, and the difference was significant (*p*<0.001). Sex-stratified analyses showed that sTM levels were higher in both the male and female subjects in the arsenic-endemic areas compared to those of the male and female subjects in the non-endemic area.

**Table 1:** Descriptive characteristics of the study populations in arsenic-endemic and non-endemic areas.

Parameters	All	Non-endemic	Arsenic-endemic	*p*-value
**Study Subjects (n)**	321	104	217	
**Sex (n)**				
Male	170	53	117	
Female	151	51	100	
**Age (years)**^a^	36.73 ± 11.36	35.02 ± 10.38	37.54 ± 11.73	0.053*
IQR	(28.00–45.00)	(27.00–40.00)	(28.00–45.00)	0.062^‡^
**BMI (kg/m^2^)**^a^	20.75 ± 3.01	21.27 ± 2.74	20.50 ± 3.11	< 0.05*
IQR	(18.66–22.40)	(19.32–22.84)	(18.32–22.34)	< 0.05^‡^
**Occupation [n, (%)]**				
**Male**				0.874^†^
Farmers	141 (82.90)	43 (81.10)	98 (83.80)	
Business	4 (2.40)	1 (1.90)	3 (2.60)	
Students	8 (4.70)	4 (7.50)	4 (3.40)	
Tailors	4 (2.40)	1 (1.90)	3 (2.60)	
^+^Others	13 (7.60)	4 (7.60)	9 (7.60)	
**Female**				0.508^†^
Housewives	137 (90.70)	46 (90.20)	91 (91.00)	
Farm workers	5 (3.30)	2 (3.90)	3 (3.00)	
Students	3 (2.00)	0	3 (3.00)	
^‡^Others	6 (4.00)	3 (5.90)	3 (3.00)	
**Education [n, (%)]**				
No formal education	177 (55.10)	59 (56.70)	118 (54.40)	0.592^†^
Primary	116 (36.10)	39 (37.50)	77 (35.50)	
Secondary	25 (7.80)	5 (4.80)	20 (9.20)	
Higher	3 (0.90)	1 (1.00)	2 (0.90)	
**Income/month (US$)**^a^	23.71 ± 7.58	23.19 ± 5.51	23.95 ± 8.39	0.403*
**DBP (mm Hg)** ^a^	75.89 ± 11.18	70.14 ± 9.59	78.64 ± 10.84	< 0.001*
**SBP (mm Hg)** ^a^	117.46 ± 17.24	110.58 ± 14.54	120.76 ± 17.49	< 0.001*
**Hypertension (n, [%])**				
Yes	32 (10.00)	2 (1.90)	30 (13.80)	< 0.01^†^
No	289 (90.00)	102 (98.10)	187 (86.20)	
**Smoking in male [n, (%)]**				
Yes	63 (37.1)	20 (37.70)	43 (36.80)	0.902^†^
No	107 (62.90)	33 (62.30)	74 (63.20)	
**Alcohol intake**	-	-	-	-
**Drinking water As (μg L^-1^)** ^a^	116.91 ± 150.17	2.33 ± 2.78	171.82 ± 155.09	< 0.001*
Range (min-max)	(0.03–546.00)	(0.03–13.17)	(0.46–546.00)	
**Hair As (μg g^-1^)** ^a^	3.88 ± 5.79	0.32 ± 0.25	5.58 ± 6.38	< 0.001*
Range (min-max)	(0.02–37.24)	(0.02–1.62)	(0.25–37.24)	
**Nail As (μg g^-1^)** ^a^	6.61 ± 6.65	1.26 ± 1.33	9.18 ± 6.66	< 0.001*
Range (min-max)	(0.15–37.42)	(0.15–8.13)	(0.53–37.42)	
**sTM (ng mL^-1^)** ^a^				
Total subjects	4.02 ± 2.12	2.84 ± 1.29	4.58 ± 2.20	< 0.001*
Male	4.40 ± 2.18	3.25 ± 1.41	4.92 ± 2.27	< 0.001*
Female	3.58 ± 1.96	2.41 ± 0.99	4.18 ± 2.07	< 0.001*
*p*-value	< 0.01*	< 0.01*	< 0.05*	

Abbreviations: As, Arsenic; BMI, Body Mass Index; IQR, Inter-quartile range. BMI was calculated as body weight (kg) divided by height squared (m^2^).

^a^ Mean ± SD.

* *p*-, ^‡^ *p*- and ^†^ *p*-values were obtained by independent sample *t*-test, Kruskal-Wallis test and Chi-square test, respectively.

^+^ Others included village doctor, carpenter, rickshaw puller, security guard and retired worker.

^‡^ Others included farmer and laborer.

### Association between arsenic exposure and sTM levels

**Fig 1:**
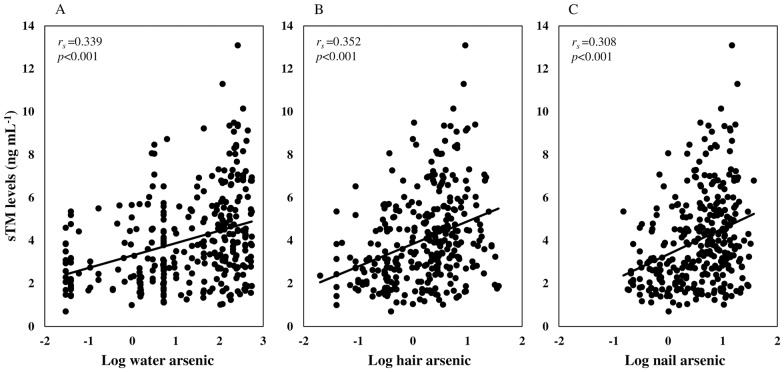
Association between arsenic exposure and serum sTM levels. Log_10_-transformed values of water (μg L^-1^), hair (μg g^-1^), and nail (μg g^-1^) arsenic concentrations were used. *r*_*s*_ and *p*-values were from Spearman correlation coefficient test.

Fig 1 shows the association between arsenic concentrations in the drinking water, hair and nails and serum sTM levels of the study populations. A significant (*r*_*s*_ = 0.339, *p*<0.001) positive association was observed between the drinking water arsenic concentrations (Fig 1A) and sTM levels. Almost similar relationships were also observed between the hair arsenic concentrations and sTM levels (*r*_*s*_ = 0.352, *p* < 0.001) (Fig 1B), and between the nail arsenic concentrations and sTM levels (*r*_*s*_ = 0.308, *p*<0.001) (Fig 1C).

### Dose-response relationship between arsenic exposure and sTM levels

**Fig 2:**
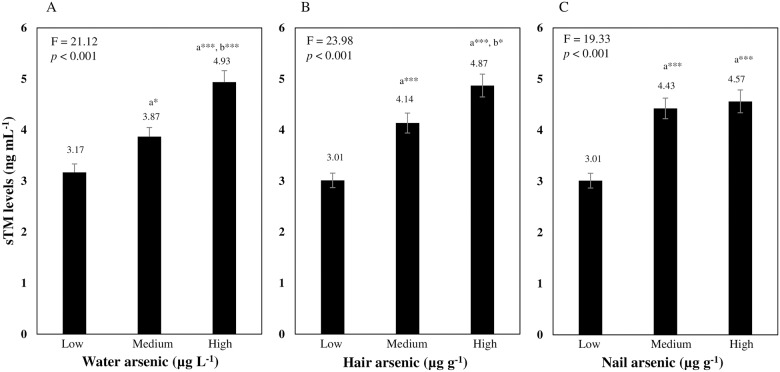
Dose-response relationship between arsenic exposure and sTM levels. Data were presented as mean ± SE. Dose-response relationship between arsenic exposure and sTM levels in one-way ANOVA was examined by F-test, followed by Bonferroni multiple comparison test between each group of exposure level. Arsenic concentrations in water: low (0.03–5.22 μg L^-1^; n = 101), medium (5.30–127.00 μg L^-1^; n = 110), and high (129.00–546.00 μg L^-1^; n = 110). Arsenic concentration in hair: low (0.02–0.62 μg g^-1^; n = 106), medium (0.64–3.36 μg g^-1^; n = 106), and high (3.40–37.24 μg g^-1^; n = 109). Arsenic concentrations in nail: low (0.15–2.12 μg g^-1^; n = 104), medium (2.14–7.45 μg g^-1^; n = 109), and high (7.45–37.42 μg g^-1^; n = 108). ^a^ and ^b,^ Significant from low and medium groups, respectively. ***p < 0.001; **p < 0.01; *p < 0.05.

Next, we checked the dose-dependent associations of arsenic exposure with serum sTM levels as shown in Fig 2. All subjects were split into tertile groups (low, medium and high) based on the subjects’ water, hair and nail arsenic concentrations. First, we checked the dose-response relationship between external exposure metric (water arsenic concentrations) and sTM levels (Fig 2A). We found that sTM levels were increased in the higher arsenic concentration gradients compared to the lower one, and the differences were significant (*p*<0.05) in medium versus low, high versus low (*p*<0.001), and high versus medium (*p*<0.001) exposure group. We then evaluated the dose-response relationship between the internal exposure metrics (hair and nail arsenic concentrations) and sTM levels (Fig 2B and 2C). Serum sTM levels were higher in the higher exposure gradients and the differences were significant in the medium versus low (*p*<0.001 for hair and nails), high versus low (*p*<0.001 for hair and nails) exposure groups of the subjects’ hair and nail arsenic concentrations. Additionally, high versus medium exposure groups of hair arsenic showed significant (*p*<0.05) difference in sTM levels.

### Multiple linear regression analyses for the associations of arsenic exposure and other variables with sTM levels

Table 2 shows the associations of arsenic exposure and other relevant demographic variables (age, sex, BMI, smoking habit and hypertension) with sTM levels through multiple linear regression analyses. The drinking water, hair and nail arsenic concentrations of the subjects showed significant (*p*<0.001 for water, hair and nail arsenic) associations with sTM levels after adjustment for age, sex, BMI, smoking habit and hypertension. Sex and hypertension showed significant association with sTM levels. Since, we found significant (*p*<0.01) association between sex and sTM levels, we further performed regression analyses stratifying the subjects into male and female groups. Arsenic exposure metrics had significant association with sTM levels in both sexes even after adjustment for age, BMI, smoking and hypertension. These results indicated that confounding effect of sex in the regression model might be due to the base line difference in sTM levels between male and female subjects which we observed in Table 1. Significant association between hypertension and sTM levels was found in all and female subjects but not in male subjects.

**Table 2:** Associations of arsenic exposure and other variables with sTM levels through multiple linear regression analyses.

Independent variables	Dependent variable sTM levels											
	All subjects^a^				Males^b^				Females^c^			
	Before adjustment		After adjustment		Before adjustment		After adjustment		Before adjustment		After adjustment	
	β (95% CI)	*p*-value	β (95%CI)	*p*-value	β (95%CI)	*p*-value	β (95%CI)	*p*-value	β (95%CI)	*p*-value	β (95%CI)	*p*-value
**Water As**	0.579 (0.403,0.754)	<0.001	0.526 (0.349,0.704)	<0.001	0.633 (0.364,0.902)	<0.001	0.638 (0.355,0.920)	<0.001	0.515 (0.294,0.736)	<0.001	0.458 (0.237,0.678)	<0.001
**Age**			0.000 (–0.019,0.020)	0.983			0.016 (–0.010,0.043)	0.220			–0.021 (–0.050,0.008)	0.161
**Sex**			0.846 (0.348,1.343)	<0.01								
**BMI**			–0.032 (–0.107,0.044)	0.407			0.053 (–0.074,0.180)	0.410			–0.083 (–0.172,0.007)	0.071
**Smoking**			–0.154 (–0.767,0.460)	0.622			–0.172 (–0.825,0.480)	0.603				
**Hypertension**			0.769 (0.010,1.527)	<0.05			–0.368 (–1.689,0.953)	0.583			1.470 (0.594,2.347)	<0.01
**Hair As**	1.050 (0.728,1.373)	<0.001	0.996 (0.673,1.319)	<0.001	0.999 (0.553,1.445)	<0.001	0.999 (0.531,1.468)	<0.001	1.168 (0.719,1.617)	<0.001	1.043 (0.601,1.485)	<0.001
**Age**			0.003 (–0.016,0.022)	0.768			0.016 (–0.010,0.042)	0.234			–0.011 (–0.040,0.017)	0.427
**Sex**			0.929 (0.434,1.424)	<0.001								
**BMI**			–0.031 (–0.107,0.044)	0.410			0.046 (–0.082,0.173)	0.481			–0.086 (–0.175,0.002)	0.056
**Smoking**			–0.136 (–0.747,0.475)	0.663			–0.162 (–0.818,0.495)	0.628				
**Hypertension**			0.791 (0.038,1.543)	<0.05			–0.379 (–1.711,0.953)	0.575			1.493 (0.635,2.351)	<0.01
**Nail As**	1.199 (0.801,1.596)	<0.001	1.094 (0.696,1.492)	<0.001	1.290 (0.690,1.890)	<0.001	1.334 (0.707,1.962)	<0.001	1.094 (0.586,1.601)	<0.001	0.951 (0.455,1.447)	<0.001
**Age**			0.005 (–0.014,0.025)	0.597			0.021 (–0.006,0.047)	0.122			–0.014 (–0.043,0.015)	0.355
**Sex**			0.904 (0.404,1.404)	<0.001								
**BMI**			–0.024 (–0.100,0.053)	0.542			0.054 (–0.074,0.182)	0.406			–0.072 (–0.163,0.018)	0.117
**Smoking**			–0.217 (–0.834,0.400)	0.489			–0.246 (–0.902,0.409)	0.459				
**Hypertension**			0.836 (0.075,1.597)	<0.05			–0.464 (–1.805,0.877)	0.495			1.596 (0.722,2.470)	<0.001

Log_10_-transformed values of arsenic concentrations were used.

^a^ Adjusted for age, sex, BMI, smoking habit and hypertension.

^b^ Adjusted for age, BMI, smoking habit and hypertension.

^c^ Adjusted for age, BMI and hypertension.

### Interaction between arsenic exposure and hypertension on sTM levels

Table 3 shows the interaction between arsenic exposure and hypertension on sTM levels through univariate regression analyses. Arsenic exposure (drinking water, hair and nail arsenic concentrations) but not hypertension showed significant association with sTM levels. Effect of interaction between arsenic exposure and hypertension on sTM levels was not significant which suggested that the effect of arsenic exposure on sTM levels was not conditional on hypertension.

**Table 3:** Interaction between exposure and hypertension on sTM levels through univariate regression analyses.

Independent variables	Dependent variable sTM levels					
	All subjects		Males		Females	
	β (95%CI)	*p*-value	β (95%CI)	*p*-value	β (95%CI)	*p*-value
**Water As**	0.568 (0.385,0.752)	<0.001	0.654 (0.377,0.931)	<0.001	0.447 (0.215,0.679)	<0.001
**Hypertension**	1.037 (–0.701,2.774)	0.241	1.321 (–2.845,5.487)	0.532	1.347 (–0.435,3.129)	0.137
Water As*Hypertension	–0.271 (–1.089,0.546)	0.514	–0.758 (–2.750,1.233)	0.453	–0.057 (–0.890,0.777)	0.894
**Hair As**	0.945 (0.613,1.278)	<0.001	0.982 (0.523,1.442)	<0.001	0.936 (0.482,1.390)	<0.001
**Hypertension**	–0.322 (–1.495,0.852)	0.590	– 1.247 (–3.730,1.236)	0.323	0.395 (–0.816,1.606)	0.520
Hair As*Hypertension	1.662 (–0.078,3.402)	0.061	1.651 (–1.737,5.039)	0.337	1.857 (–0.028,3.742)	0.053
**Nail As**	1.077 (0.664,1.491)	<0.001	1.267 (0.647,1.886)	<0.001	0.858 (0.340,1.377)	<0.01
**Hypertension**	–0.302 (–1.862,1.257)	0.703	– 2.654 (–7.232,1.924)	0.254	0.417 (–1.105,1.939)	0.589
Nail As*Hypertension	1.153 (–0.558,2.864)	0.186	2.551 (–2.087,7.189)	0.279	1.337 (–0.408,3.082)	0.132

Log_10_-transformed values of arsenic concentrations were used.

* Interaction was calculated by multiplying the arsenic concentrations and hypertension.

### Association between sTM levels and circulating molecules of CVD risk

S1 Table shows the association between serum sTM levels and plasma circulating molecules involved in atherosclerosis. Previously in the same populations, we measured several circulating biomarkers related to atherosclerosis, and reported that chronic exposure to arsenic was positively associated with ICAM-1, VCAM-1 levels, and negatively associated with HDL-C [18]. Association between arsenic exposure and sTM levels observed in this study led us to analyze the relationship of sTM levels with other circulating markers related to the risk of CVD that we reported in our previous study [18]. All subjects (n = 321) in present study were overlapped with the subjects used for the measurement of ICAM-1, VCAM-1 and HDL-C in our previous study. Intriguingly, we found that sTM levels had a significant negative (*r*_*s*_ = –0.205, *p*< 0.001) association with HDL-C and significant positive associations with ICAM-1 (*r*_*s*_ = 0.299, *p*<0.001) and VCAM-1 (*r*_*s*_ = 0.253, *p*<0.001).

## Discussion

Because of the central role of the endothelium in the development and clinical course of atherosclerosis, endothelial function testing may serve as a useful biomarker of atherosclerosis. Soluble TM (sTM) has been shown to be a specific and a stable marker for endothelial cell damage or dysfunctions [23, 30,38]. In this study, we found that serum sTM levels of the subjects in arsenic-endemic areas were significantly (*p*<0.001) higher than those of the subjects in non-endemic areas (Table 1). Drinking water, hair and nail arsenic concentrations of the subjects were positively (water arsenic: *r*_*s*_ = 0.339, *p* <0.001, hair arsenic: *r*_*s*_ = 0.352, *p*<0.001, nail arsenic: *r*_*s*_ = 0.308, *p* <0.001) associated with serum sTM levels (Fig 1). Arsenic exposure levels also showed a dose-response relationship with serum sTM levels (Fig 2).

Upon endothelial cell damage, the TM produced in endothelial cell is degraded by intracellular proteases and is released into the blood, where it becomes sTM, and is excreted in urine. Since endothelial cell production, function and the severity of the damage are inferred by measuring the concentration of sTM in the blood by simple ELISA technique, sTM is useful as a marker of vascular endothelial cell damage [23]. Although most of the previous studies reported the positive association between sTM and CVD [30, 39,40] but some studies did not find association [41–43]. These contradictions may cause ambiguity in interpreting the pathophysiological significance of elevated levels of sTM observed in arsenic-exposed individuals. To eliminate this ambiguity, we further tested the associations of elevated levels of sTM with the other blood markers of CVD risk related to the chronic exposure to arsenic as shown in our previous study [18]. The same blood specimens of the subjects in the previous study were used to determine the serum sTM levels. Intriguingly, we found that elevated levels of serum sTM levels were inversely (*r*_*s*_ = –0.205, *p*< 0.001) associated with the levels of circulating HDL-C, which is often considered as “good” lipoprotein for its ability to transport cholesterol from arterial walls to liver for degradation (S1 Table). Adhesion molecules (ICAM-1 and VCAM-1) expressed by endothelial cells are involved in the recruitment and transendothelial migration of leucocytes leading to the initiation of atherosclerosis [44,45]. Soluble TM (sTM) levels were found to be positively associated with circulating ICAM-1 (*r*_*s*_ = 0.299, *p*<0.001) and VCAM-1 (*r*_*s*_ = 0.253, *p*<0.001) levels (S1 Table). All these results suggest that as a consequence of endothelial dysfunction, elevated levels of sTM may be an indicator of future risk of arsenic-induced CVD.

It should be noted, however, that the significance of sTM may be different in healthy subjects and in patients with CVD. In healthy individuals who do not have CVD, the high concentrations of sTM have been reported to be associated with the decreased risk of coronary heart disease [42]. This is because in healthy individuals, the concentration of sTM reflects the quantity of TM expressed on the endothelial cell surface and increased TM expression may raise the sTM levels in circulation. High concentration of sTM in healthy individuals without CVD may indicate a low pro-thrombotic state and decrease the rate of coronary heart diseases. Whereas, in CVD, the elevated levels of sTM reflects the degree of vascular damage rather than the expression level of TM on endothelium surface [30, 39, 40].

Generally, high levels of circulating triglyceride (TG), total cholesterol (TC) and low density lipoprotein cholesterol (LDL-C), and low level of high density lipoprotein cholesterol (HDL-C) are considered for CVD risk clinically. In our previous study [18] on the same population that we used for this study, however, we found that TG, TC and LDL levels were decreased with the increasing concentrations of arsenic. Except the association with TG, the other two negative associations were significant. We also found that HDL-C levels were decreased significantly with the increasing concentrations of arsenic which indicated that only circulating HDL-C level but not the other three lipid markers (TG, TC and HDL-C) may be useful for the assessment of CVD risk in the chronic arsenic-exposed individuals. Only one lipid marker may not be sufficient for the assessment of future risk of CVD in the chronic arsenic-exposed people. Therefore, more reliable blood markers that can be sensitive to early stage of atherosclerotic event are necessary. Precise nature of dose-response relationship between chronic exposure to arsenic and serum sTM levels observed in this study (Fig 2) suggest that elevated serum sTM may have potential to be a marker of arsenic-induced endothelial dysfunction/damage as an early event of CVD.

Several studies have considered age, sex, BMI, smoking and other characteristics as possible confounders for sTM [46–51]. Dohi et al (2003) reported that sTM levels were higher in hypertensive patients than those of normotensive control group [52]. Therefore, in multiple-linear regression analyses (Table 2), we considered age, sex, BMI, smoking and hypertension as variables, and we found that sex and hypertension showed significant association with sTM levels. The main reason for obtaining significant association between sex and sTM in regression analyses was probably because of the base level difference in sTM levels between male and female subjects as observed in Table 1. We found that the average sTM level in males was significantly higher than those in females. This result was in good agreement with the previous report that showed the significant differences of sTM levels between male and female groups [46]. Hypertension was found to be a significant confounders for sTM levels in all and female subjects. However, in univariate regression analyses as shown in Table 3, we did not find any significant interaction effect between arsenic exposure and hypertension on sTM levels which suggested that the relationship between arsenic exposure and sTM levels was not dependent on hypertension (Table 3). In regression analysis, no significant associations between sTM levels and age, BMI or smoking habits were observed (Table 2). Previous studies showed inconsistent associations between these variables and sTM levels [47, 48, 51]. In our study, we did not include any older aged (>60 years) subject. IQR of the age and BMI of our subjects were 28.00–45.00 and 18.66–22.40, respectively (Table 1). Furthermore, the majority (62.90%) of the male subjects was not smokers and no females were smokers (Table 1). The narrow ranges of age and BMI, and the smaller number of smokers may explain why we did not observe any confounding effects of those variables on sTM levels.

The strengths of this study include, first, this study was conducted on well-defined study subjects based on three kinds of arsenic exposure metrics. Second, associations of sTM were found across the three kinds of arsenic exposure metrics that may reduce the bias in misclassification. Third, wide variation of arsenic concentrations (Table 1) of the study subjects which showed a dose-dependent association with sTM (Fig 2). This dose-dependent relationship between arsenic exposure and sTM observed in this study suggest that sTM may have potential to be an indicator of chronic arsenic exposure-related vascular damage.

This study had some potential limitations that warranted further discussion. Several studies have reported the presence of other metals along with arsenic in the drinking water in Bangladesh [53,54]. In our study, we did not consider the effects of other metals on sTM levels that could be present in the ground water of Bangladesh. If other accompanying chemical agent in drinking water could also be responsible for the observed association between arsenic exposure and sTM levels, the chemical would follow the same concentration gradient as arsenic in the drinking water. This is unlikely; however, we cannot completely exclude the possibility that the effects of other metals or any other confounders are involved in the increase in sTM levels. Therefore, more detailed examination of the chemical components in the drinking water may be required in a future study. Another limitation was that the results of our study could not show the cause-effect relationship between arsenic exposure and sTM levels. Therefore, like all association studies, the findings need to be replicated through a long-term cohort study.

Measuring endothelial function in humans is potentially useful for prognosis, and assessment of severity and future risk of CVD in asymptomatic patients, and for measuring drug efficacies [23,55]. We and other groups have reported the pro-atherogenic effects of chronic exposure to arsenic [18, 56,57]. Endothelial dysfunction has been proposed to be an early event of atherosclerotic process [58,59]. Early diagnosis of future risk of CVD in the people living in arsenic-endemic areas may be useful strategies to decrease the morbidity and mortality from chronic exposure to arsenic. Hence, measuring serum sTM in chronic arsenic-exposed individual may be useful for understanding and assessing the risk of CVD.

## Conclusion

This study shows that serum sTM levels are significantly higher in the population in arsenic-endemic areas than those of the population in non-endemic area. Subjects’ drinking water, hair and nail arsenic concentrations also show positive and dose-dependent relationships with sTM levels. These results show that chronic exposure to arsenic has mild to moderate association with sTM levels.

## Supporting information

S1 Table Association between sTM levels and circulating molecules of CVD risk. *r*_*s*_ and *p*-values were from Spearman correlation coefficient test.
(DOC)
