# Supplementary material for: Association between arsenic exposure and soluble thrombomodulin: A cross sectional study in Bangladesh
Source: PLoS One. 2017 Apr 11;12(4):e0175154. doi: 10.1371/journal.pone.0175154 (PMC5388467; doi:10.1371/journal.pone.0175154)
Supplement: S1 Table — rs and p-values were from Spearman correlation coefficient test. (DOC) [file pone.0175154.s001.doc]

**S1 Table** Association between sTM levels and circulating molecules of CVD risk.

|  | sTM levels | |
| --- | --- | --- |
|  | Correlation coefficient (*r_s_*) | *p*-value |
| HDL-C | –0.205 | <0.001 |
| ICAM-1 | 0.299 | <0.001 |
| VCAM-1 | 0.253 | <0.001 |

*r_s_* and *p*-values were from Spearman correlation coefficient test.
